# No evidence that herpes zoster is associated with increased risk of dementia diagnosis

**DOI:** 10.1002/acn3.51525

**Published:** 2022-02-16

**Authors:** Charlotte Warren‐Gash, Elizabeth Williamson, Suhail I. Shiekh, James Borjas‐Howard, Neil Pearce, Judith M. Breuer, Liam Smeeth

**Affiliations:** ^1^ Department of Non‐Communicable Disease Epidemiology London School of Hygiene & Tropical Medicine Keppel Street London WC1E 7HT; ^2^ Department of Medical Statistics London School of Hygiene & Tropical Medicine Keppel Street London WC1E 7HT; ^3^ Department of Haematology University Medical Centre Groningen, University of Groningen Groningen The Netherlands; ^4^ Institute of Child Health University College London Gower Street London WC1E 6BT

## Abstract

**Objective:**

To investigate whether herpes zoster (HZ) was associated with subsequent increased risk of dementia diagnosis.

**Methods:**

We conducted a historical cohort study using primary care electronic health records from the Clinical Practice Research Datalink in the United Kingdom. Individuals with incident HZ aged ≥40 years from 2000 to 2017 were matched with up to four individuals without HZ by age, sex, primary care practise and calendar time. The primary outcome was a new diagnosis of all‐cause dementia. We used the Cox proportional hazards regression adjusting for demographic, lifestyle and clinical confounders to assess any association between HZ and dementia. We investigated interactions with sex, frailty index and antiviral treatment and conducted various sensitivity analyses.

**Results:**

The cohort comprised 177,144 individuals with HZ and 706,901 matched unexposed individuals (median age 65 years (IQR 55.1–75.0), 40% male) followed for a median duration of 4.6 years (IQR 2.0–8.1). In total, 26,585 (3%) patients had an incident dementia diagnosis recorded and 113,056 patients died (12.8%). HZ was associated with a small reduction in dementia diagnosis (adjusted HR 0.92 (95% CI 0.89–0.95)), occurring predominantly in frail individuals and females. For patients who were fit (578,115, 65%), no association was seen (adjusted HR 0.97, 95% CI 0.92–1.02). There was no association between HZ and a composite outcome of dementia or death (adjusted HR 1.00, 95% CI 0.99–1.02). Dementia risk did not vary by prescription of antiviral agents. Sensitivity analyses showed consistent results.

**Interpretation:**

HZ was not associated with increased dementia diagnosis in a UK primary care‐based cohort.

## Introduction

The global burden of dementia is rising, with the number of people living with dementia projected to reach 131.5 million in 2050 compared to 46.8 million in 2015.^1^ Driven by increases in life expectancy among low‐ and middle‐income countries, this will have a major impact upon disability, dependence and quality of life, especially in settings where access to care and support services is limited.^2^ Only around 40% of dementia cases are potentially preventable by action on known modifiable risk factors.^3^ Combined with the persistent failure of dementia treatment trials, this has prompted renewed urgency in the search for tractable dementia risk factors.

Infections may exacerbate or accelerate progression of a range of underlying diseases.^4^, ^5^, ^6^, ^7^ In the brain, a dysregulated microglial response to injurious stimuli may disrupt usual sentinel and housekeeping pathways, leading to ongoing neuroinflammation and neuronal damage. Dysregulation of host immune pathways is implicated both in Alzheimer's disease pathogenesis, for example, through persistent amyloid beta deposition resulting from chronic interaction with microglia, and in the tauopathies, which are associated with accumulation of pro‐inflammatory microglia, toxic insoluble tau and neuronal loss.^8^ Although precise mechanisms remain controversial, the effect of a dysregulated immune response to HIV infection contributes to HIV‐associated neurocognitive disorders.^9^ While other chronic viral infections are likely to trigger microglial responses, whether, when and in whom such infections might affect progression to clinical dementia is unknown.

The incidence of herpes zoster (HZ), a painful, blistering, dermatomal rash that occurs due to varicella zoster virus reactivation, rises sharply with age as cell‐mediated immunity declines. HZ may lead to localised post‐herpetic neuralgia, as well as a range of, albeit uncommon, acute ophthalmic, visceral and neurological complications such as stroke.^10^ It is less clear whether HZ has other long‐term sequelae including effects on neurodegeneration. A small propensity score matched cohort from Taiwan (*N* = 3384) demonstrated a threefold increase in dementia risk after ophthalmic zoster,^11^ but findings from two matched cohort studies using health insurance claims data from Taiwan and Korea suggested that any increased risk of dementia associated with HZ is likely to be modest (11%–12%).^12^, ^13^ Recent results from a Korean case control study showed no evidence for an association.^14^ Subgroup analyses from a multi‐centre Northern European cohort focussed on antiviral medication use after herpes viruses also failed to support these findings.^15^ It is unclear whether differences between populations or within population subgroups could explain the contrasting findings. If a robust, replicable effect were present among certain groups, this could have important implications for HZ vaccination and treatment policy, especially in settings of high infectious burden.

We therefore aimed to investigate whether HZ was associated with subsequent dementia diagnosis overall and among specific population subgroups using routine clinical health data from the United Kingdom.

## Methods

### Data sources

We used electronic health records data from the Clinical Practice Research Datalink (CPRD). The CPRD Gold database contains anonymised primary care records from approximately 7% of the UK population who are broadly representative of the general population by age, sex and ethnicity.^16^ Longitudinal records are generated through routine care and include demographic information, clinical events such as diagnoses and symptoms, prescriptions, investigations, referrals and some lifestyle factors. Data are recorded by general practice staff using Read codes, a hierarchical clinical classification system, along with numerical data on additional clinical measures such as height, weight and blood pressure, and prescriptions issued by the GP with a product name and British National Formulary code. Health information received by the practise from other sources such as secondary care is also coded and entered into the patient record. We also used linked small area deprivation data defined by the index of multiple deprivation (IMD) at general practise level. IMD is a relative measure of deprivation calculated for lower‐layer super output areas, which is based upon seven domains (income; employment; education, skills and training; health deprivation and disability; crime; barriers to housing and services; living environment deprivation).^17^

### Study design

We conducted a historical cohort study using a matched design. Eligible patients identified as having incident herpes zoster diagnosed during the study period were matched to up to four eligible patients who had not been diagnosed with herpes zoster. Patients were matched on age (within 1 year), sex, general practise and calendar time. Exposure was time‐updated: patients who were selected as an unexposed match could subsequently enter the study as an exposed patient if they developed herpes zoster; their follow‐up as an unexposed match was censored at diagnosis of herpes zoster.

### Study population and follow up

Patients were eligible for inclusion if they were aged 40 years or over and had no history of prior dementia or prior herpes zoster. The study period ran from 1 January 2000 to 31 December 2017. Eligible follow‐up time began at the latest of: 1 January 2000, 40th birthday, or having 12 months of eligible follow‐up time within CPRD (when the patient was registered at the practise and practise data were considered to meet acceptable quality standards). Patients were required to be registered with the practise for at least 12 months prior to study entry to prevent historical recording of diagnoses at registration. Follow up began at the index date, which was the date of herpes zoster diagnosis for exposed patients or the date of diagnosis of the exposed match for unexposed patients. Follow‐up ended at the earliest of: dementia diagnosis, death, end of CPRD follow‐up (transfer out of the general practise or last collection date from the practise) or 31 December 2017.

### Exposure definition

Exposure was defined as incident herpes zoster at any site ascertained through diagnosis and referral codes in GP records with the date of the first record taken as the index date. In secondary analyses, we investigated the effect of ophthalmic zoster versus zoster at any other site. Ophthalmic zoster was defined either by the presence of a specific code for ophthalmic zoster in the year following first diagnosis, or by a non‐specific zoster code plus a diagnosis of, or treatment for, acute eye infection (such as keratitis or conjunctivitis) within 2 weeks of zoster onset, or from records of first‐ever‐specific chronic eye conditions known to be associated with zoster (such as, conjunctival scarring or episcleritis), within 3 months of zoster using an approach defined in previous studies.^18^ We also explored any association between antiviral treatment for zoster, defined as a prescription for an antiviral agent in the 7 days following the index date, and dementia.

### Outcome definition

The primary outcome was incident all‐cause dementia defined using diagnostic and referral codes in primary care records, with the date taken as the date of first record. In a sensitivity analysis, we expanded the outcome definition to include administrative as well as diagnosis and referral codes, taking the first of any type of code as dementia date. In this analysis, this wider definition was also used to exclude patients with prior dementia. Secondary outcomes were dementia subtypes (Alzheimer's disease, vascular dementia, other specified dementia, mixed/unspecified dementia). For individuals whose records contained more than one specified dementia subtype, the subtype was recoded to ‘mixed’.

### Covariate definitions

Potential confounders were identified in primary care electronic health records used Read codes based upon previous literature and a priori knowledge of risk factors for exposure and outcome. Codelists are available on LSHTM Data Compass (https://doi.org/10.17037/DATA.00002672). Age, sex, general practise and calendar time were matching factors and also potential confounders. Year of study entry, when included in models, was grouped into 2000–2003, 2004–2006, 2007–2009, 2010–2013, 2013–2017. Additional confounders defined at study entry were: frailty, as measured by the electronic frailty index (eFI),^19^ grouped into four categories of increasing frailty, grouped frequency of consultations with the general practise in the past 3 years as a measure of health seeking behaviour, harmful alcohol use, body mass index (BMI; grouped into underweight (<18.5), normal (18.5–25), overweight (25–30) and obese (>30) using the record closest to the index date), smoking (never, current and former), uncontrolled diabetes (measure of glycated haemoglobin of 7.5% or above in the last 2 years) and prior diagnosis of: chronic kidney disease, asthma, autoimmune disease, chronic obstructive pulmonary disease, depression in the last 2 years, diagnosed hypertension, ischaemic heart disease, liver disease, stroke, traumatic brain injury and herpes simplex virus. Immunosuppression was determined through diagnoses, referrals and prescriptions (HIV, organ transplant or conditions leading to permanent immune deficiency or aplastic anaemia ever diagnosed, haematological malignancy or bone marrow transplant within the last 2 years, impaired cell‐mediated immunity, chemotherapy or biologics within the last year, or two prescriptions of other oral steroids in the last year). Potential confounding by ethnicity (Black, White, South Asian, Mixed/Other) was assessed in sensitivity analysis only. Socio‐economic status at practise level was measured by quintile of the 2015 index of multiple deprivation (IMD).

### Statistical analysis

We first described the characteristics of the cohort by exposure group at study entry. Age‐ and sex‐adjusted rates of incident dementia were calculated by exposure group. We fitted Cox proportional hazards regression models using age as the underlying timescale to estimate cause‐specific hazards of dementia among those with and without HZ exposure. The initial model was adjusted for age (as the timescale) and sex and stratified by practise. Fully adjusted models additionally included the pre‐specified individual‐level confounders detailed above. Robust standard errors clustered by patient were used to account for patients potentially appearing as both exposed and unexposed. The proportional hazards assumption was assessed graphically and using tests based on Schoenfeld residuals. Violations of the assumption were handled via stratified Cox models, if occurring in confounder variables.

We carried out several sensitivity analyses to explore missing covariate data on BMI and smoking, and compared results with the primary complete case analysis. These were: (i) we removed BMI and smoking from the primary analysis model; (ii) we assumed that patients missing smoking information were non‐smokers and patients missing BMI data had a normal BMI; (iii) we imputed missing data for BMI and smoking using chained equations under the missing at random assumption. Ten imputed datasets were created. Continuous BMI was imputed using predictive mean matching, with five nearest neighbours, and smoking using a multinomial logistic regression. Imputation was performed separately by exposure group. Imputation models included the Nelson‐Aalen estimate of cumulative hazard for dementia, the indicators for death and dementia, as well as the exposure and all potential confounders. Cause‐specific Cox models were fitted in each imputed dataset; estimates were combined using Rubin's rules. Due to the large proportion of missing ethnicity data (59%), we did not include it in the primary model. In other sensitivity analyses, we: (iv) carried out a complete case analysis with ethnicity added to our primary analysis model; (v) imputed ethnicity assuming that those with missing ethnicity were White.

Additional sensitivity analyses included (vi) exploring biases resulting from differential follow up in the exposed and unexposed by censoring follow up within a matched set at the first of: the end of the exposed patient's follow‐up, or the last of the end of the unexposed patients' follow‐up; (vii) exploring possible reverse causation by excluding the first year of follow up. Entire matched sets were excluded from this analysis if the exposed patient experienced the outcome within the first year or all unexposed matches experienced the outcome within the first year. Similarly, the first 2, 3 and 4 years of follow up were excluded; (viii) expanding the outcome definition to increase its sensitivity, as the primary outcome was likely to have high specificity but lower sensitivity. First, matched sets were excluded if the exposed patient had a history of dementia using the wider definition or all controls had a history of dementia. Second, the outcome was also defined using the wider definition. These two analyses were undertaken sequentially.

Cause‐specific hazards of death and the composite of death or dementia were estimated using the primary analysis approach described above, censoring follow up at the competing event. We plotted cumulative incidence functions under exposure and no exposure by fitting cause‐specific hazards models for death and dementia using Royston Parmar smooth parametric proportional hazards models and marginalising over the cause‐specific hazards.

Secondary analyses included restricting the exposure definition to ophthalmic zoster only. In this analysis, matched sets were excluded if the exposed patient did not have ophthalmic zoster. We also repeated analyses for the secondary outcomes Alzheimer's disease, vascular dementia, mixed or unspecified dementia and other specified causes, censoring if a different dementia subtype occurred. In other secondary analyses, we explored interactions between HZ and sex, frailty and receipt of antivirals post‐exposure.

Finally, following evidence of non‐proportionality, log‐logistic model and generalised gamma models were also fitted, which do not make the proportional hazards assumption. Model fit was compared using the AIC. Analyses were carried out using Stata version 16 (StataCorp. 2017. *Stata Statistical Software: Release 16*. College Station, TX: StataCorp LLC).

### Ethical approval

This study was approved by the Independent Scientific Advisory Committee of the CPRD (18_134R) and the LSHTM ethics committee (15991).

## Results

### Description of study population

**Figure 1:**
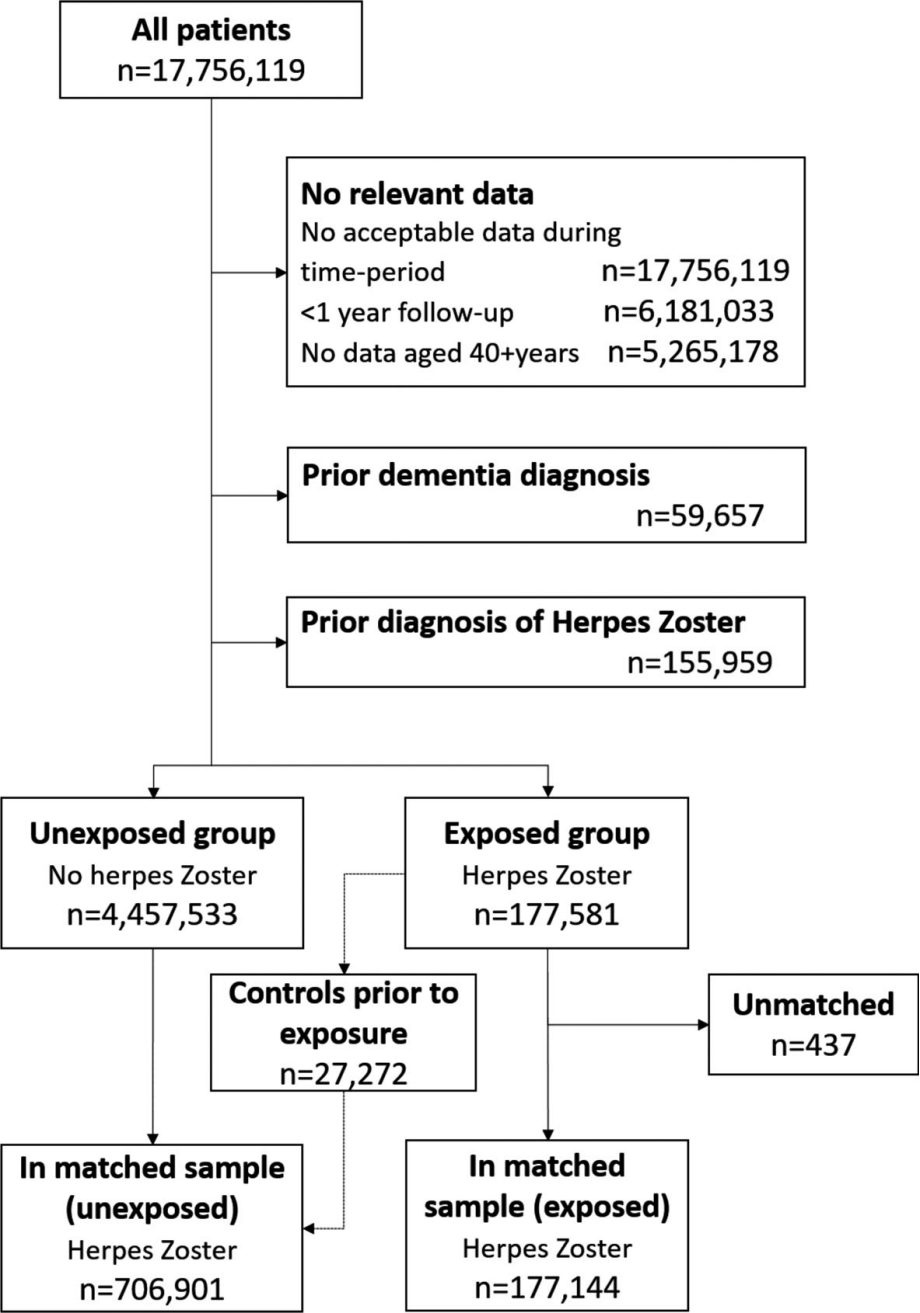
Flowchart of study population.

Figure 1 shows the flowchart of patients included in the study. Of 177,581 eligible patients with an incident HZ diagnosis, 437 (0.3%) were unable to be matched. Four unexposed matches were found for most exposed patients. For 296 sets, three matches were found, for 283 sets, two matches were found and for 271 sets, only one match was identified. The final matched cohort included 177,144 exposed patients and 706,901 matched unexposed patients.

Table 1 shows baseline characteristics of included patients by exposure status. While the age, sex and practise deprivation quintile distributions were similar due to the matched design, the HZ‐ exposed group had a slightly higher rate of primary care consultations (mean annual consultation rate 10.9 vs. 8.7) and were more likely to be classified as frail than the unexposed group (40.7% vs. 33.1%). BMI distribution and smoking rates were similar across exposure groups, but the unexposed had more missing data (8.8% vs. 5.4%; 2.9% vs. 0.6%), perhaps reflecting lower contact with health services. The HZ‐exposed group generally had a slightly higher proportion of comorbidities, with the exception of traumatic brain injury and stroke. Only 5% of HZ cases were identified as ophthalmic zoster, with site unspecified in 95% of cases. Most exposed patients (63%) received antivirals in the week following diagnosis.

**Table 1:** Baseline characteristics by exposure group.

Characteristic	No Herpes zoster	Herpes zoster
	*N* = 706,901	*N* = 177,144
Years of follow up:		
Mean (SD)	5.4 (4)	5.5 (4.1)
Median (25th, 75th ptile)	4.5 (2,8)	4.7 (2.1,8.3)
Age (years):		
Mean (SD)	65.1 (12.8)	65.1 (12.9)
Median (25th, 75th ptile)	65 (55.1,74.9)	65 (55.1,75)
Grouped age:		
40‐ < 50	99,866 (14.1)	24,967 (14.1)
50‐ < 60	161,779 (22.9)	40,444 (22.8)
60‐ < 70	184,997 (26.2)	46,163 (26.1)
70‐ < 80	160,827 (22.8)	40,320 (22.8)
80+	99,432 (14.1)	25,250 (14.3)
Male	282,061 (39.9)	70,690 (39.9)
Annual consultation rate		
Median (25th, 75th ptile)	6.3 (2.9,11.7)	8.3 (4.3,14.3)
Consultation rate (contacts/yr):		
< 10	482,332 (68.2)	103,060 (58.2)
10‐ < 20	159,423 (22.6)	50,184 (28.3)
20‐ < 30	43,054 (6.1)	15,447 (8.7)
30+	22,092 (3.1)	8453 (4.8)
Frailty Index (eFI)		
Median (25th, 75th ptile)	0.1 (0,0.2)	0.1 (0.1,0.2)
Frailty Index (eFI) group		
Fit	473,020 (66.9)	105,095 (59.3)
Mild frailty	160,726 (22.7)	47,729 (26.9)
Moderate frailty	56,356 (8.0)	18,445 (10.4)
Severe frailty	16,799 (2.4)	5875 (3.3)
BMI		
Mean (SD)	27.2 (5.3)	27.2 (5.3)
Median (25th, 75th ptile)	26.4 (23.5,29.9)	26.4 (23.6,29.9)
BMI category:		
Underweight	11,594 (1.6)	2884 (1.6)
Normal Weight	231,470 (32.7)	59,262 (33.5)
Overweight	243,383 (34.4)	63,878 (36.1)
Obese	157,956 (22.3)	41,483 (23.4)
Missing	62,498 (8.8)	9637 (5.4)
Smoking status:		
No	264,121 (37.4)	66,004 (37.3)
Yes	155,237 (22.0)	35,960 (20.3)
Ex	267,329 (37.8)	74,081 (41.8)
Missing	20,214 (2.9)	1099 (0.6)
Harmful alcohol use	24,601 (3.5)	6071 (3.4)
Ethnicity		
White	271,882 (38.5)	71,625 (40.4)
South Asian	6302 (0.9)	1376 (0.8)
Black	3485 (0.5)	559 (0.3)
Other	2530 (0.4)	615 (0.3)
Mixed	946 (0.1)	194 (0.1)
Unknown	421,756 (59.7)	102,775 (58.0)
Deprivation group:		
1 (least deprived)	138,157 (19.5)	34,604 (19.5)
2	121,460 (17.2)	30,434 (17.2)
3	144,900 (20.5)	36,320 (20.5)
4	142,616 (20.2)	35,736 (20.2)
5 (most deprived)	159,768 (22.6)	40,050 (22.6)
Prior history of:		
Asthma	87,768 (12.4)	26,956 (15.2)
COPD	63,536 (9.0)	19,931 (11.3)
Diabetes	75,524 (10.7)	20,725 (11.7)
Uncontrolled diabetes (2 years)	32,671 (4.6)	9271 (5.2)
Hypertension (diagnosed)	232,748 (32.9)	62,436 (35.2)
Ischaemic heart disease	117,646 (16.6)	34,429 (19.4)
Stroke	25,152 (3.6)	6595 (3.7)
Traumatic brain injury	5310 (0.8)	1454 (0.8)
Immunosuppressed	45,957 (6.5)	19,623 (11.1)
Autoimmune disease	53,030 (7.5)	18,015 (10.2)
Liver disease	4820 (0.7)	1359 (0.8)
Chronic kidney disease	51,882 (7.3)	15,181 (8.6)
Depression (2 years)	14,310 (2.0)	4316 (2.4)
HSV	23,607 (3.3)	8535 (4.8)
Details of exposure		
Antivirals (in 7 days)	186 (<1)	110,997 (62.7)
Site of Zoster:		
Ophthalmic		8775 (5.0)
Non‐truncal (exclud. HZO)		984 (0.6)
Site unspecified		167,385 (94.5)
Outcome events:		
Death	87,371 (12.4)	25,685 (14.5)
Dementia of which (%) of dementia)	21,213 (3.0)	5372 (3.0)
Alzheimer's	6571 (31.0)	1674 (31.2)
Vascular	4876 (23.0)	1324 (24.7)
Other specified	808 (3.8)	203 (3.8)
Mixed/ unspecified	8958 (42.2)	2171 (40.4)
Dementia (wider definition)^a^	22,902 (3.2)	5839 (3.3)

a Smaller denominator; matched sets discarded if the exposed or all unexposed patients had pre‐study dementia by this definition.

Among HZ patients, 25,685 (14.5%) died during follow up and 5372 (3%) experienced incident dementia during follow‐up compared to 87,371 (12.4%) who died and 21,213 (3%) who experienced incident dementia in the unexposed group. Follow‐up periods were similar between groups (median 4.7 years in the exposed v 4.5 years in the unexposed).

### Dementia incidence rates

**Figure 2:**
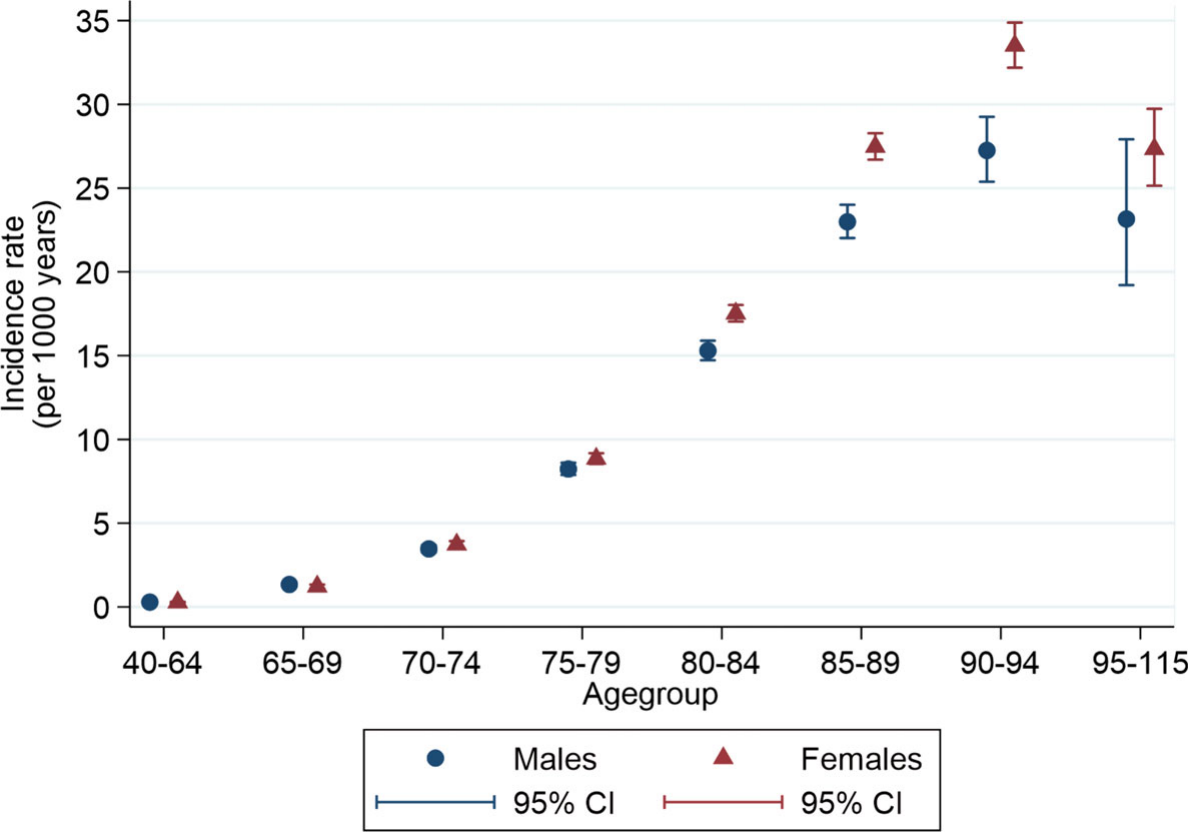
Incidence rates of dementia by age and sex. [Colour figure can be viewed at wileyonlinelibrary.com]

**Figure 3:**
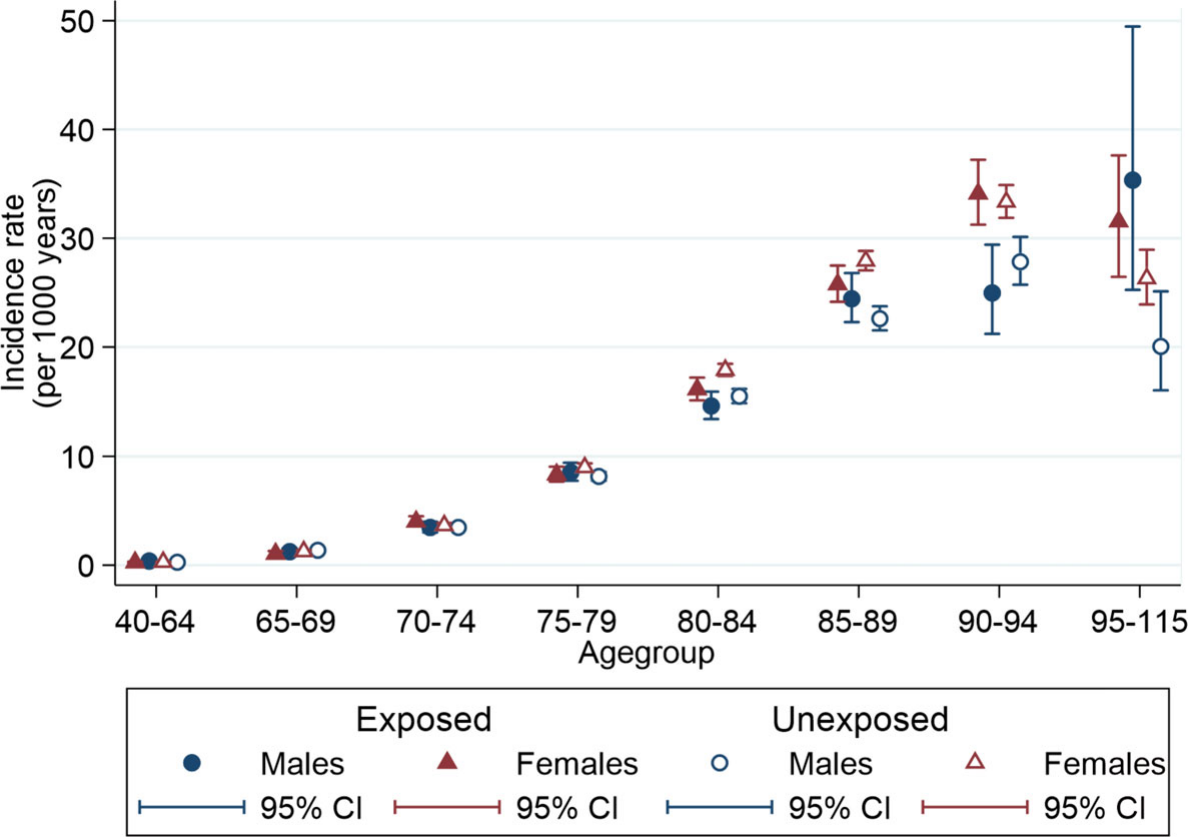
Incidence rates of dementia by age, sex and herpes zoster. [Colour figure can be viewed at wileyonlinelibrary.com]

The overall dementia incidence rate was 5.58 (95% CI 5.51–5.65) per 1000 person years. Figure 2 shows estimated incidence rates of dementia by age and sex. As expected, incidence rates increase steeply with age, before declining among people aged 95 years or more. Females had a higher dementia incidence than males apparent from 80 years and over. Figure 3 further stratifies by HZ status and shows little difference in age‐ and sex‐stratified dementia incidence rates between patients with and without HZ until estimates diverge in the oldest (>95 years) age group.

### Association between HZ and incident dementia and sensitivity analyses

Findings from the primary analysis and sensitivity analyses are shown in Table 2. HZ was not associated with incident dementia in an age‐, sex‐ and practice‐adjusted model (HR 0.97, 95% CI 0.94–1.00). Further adjustment for all confounders gave an adjusted HR of 0.92, 95% CI 0.89–0.95. Sensitivity analyses designed to deal with missing covariate data made little difference to the effect estimates: removing BMI and smoking from the model gave very similar estimates to both single and multiple imputation of these variables as well as single imputation of ethnicity data. Adjusting for ethnicity in a complete case analysis resulted in almost identical estimates to the primary analysis (adjusted HR 0.92, 95% C.I 0.88–0.97). Both truncating follow up (adjusted HR 0.90, 95% CI 0.87–0.93) and delaying entry to the study (adjusted HR 0.89, 95% CI 0.86–0.92 for 1 year delay) resulted in estimates that were slightly further from the null value. Using a broader definition of dementia as the outcome did not materially alter results (adjusted HR 0.92, 95% CI 0.89–0.95).

**Table 2:** Estimated hazard ratios from Cox models for primary analysis and sensitivity analyses.

Type of analysis	HR (95% CI)
Primary analysis	
Sex‐, age‐ and GP‐adjusted Fully adjusted model^a^	0.97 (0.94, 1.00) 0.92 (0.89, 0.95)
**Sensitivity analyses**	
(i) No adjustment for BMI and smoking (ii) Single imputation (BMI/smoking) (iii) Multiple imputation (BMI/smoking) (iv) Additional adjustment for ethnicity (v) Single imputation (BMI/smoking/ethnicity) (vi) Delayed entry • By 1 year • By 2 years • By 3 years • By 4 years (vii) Truncated follow up (viii) a) Excluding those with pre‐study dementia (wider definition)b) Outcome definition via wider definition	0.93 (0.90, 0.96) 0.93 (0.90, 0.96) 0.93 (0.90, 0.96) 0.92 (0.88, 0.97) 0.93 (0.90, 0.96) 0.89 (0.86, 0.92) 0.87 (0.84, 0.90) 0.86 (0.83, 0.89) 0.86 (0.82, 0.90) 0.90 (0.87, 0.93) 0.92 (0.89, 0.94) 0.92 (0.89, 0.95)

^a^ Adjusted for sex, age, practice, year of study entry, frailty index, prior consultation rate, harmful alcohol use, BMI, smoking, chronic kidney disease, asthma, autoimmune disease, COPD, depression, hypertension, ischaemic heart disease, severe immunosuppression, liver disease, stroke, traumatic brain injury, herpes simplex, uncontrolled diabetes.

### Competing risks analysis: association between HZ and dementia, death and dementia or death

Table 3 shows estimated hazard ratios from the primary analysis model, for outcomes of dementia, death and their composite. There was a small increase in risk of death among individuals with HZ (adjusted HR 1.02, 95% CI 1.01–1.04) while the composite outcome of death and dementia showed no association with HZ (adjusted HR 1.00, 95% CI 0.99–1.02).

**Table 3:** Estimated HRs for the three outcomes of dementia, death and their composite.

	HR (95% CI)		
Variable	Dementia	Death	Composite
Herpes Zoster	0.92 (0.89, 0.95)	1.02 (1.01, 1.04)	1.00 (0.99, 1.02)
Male	0.90 (0.87, 0.92)	1.49 (1.47, 1.51)	1.35 (1.33, 1.37)
Year of study entry:			
2000–2003	0.94 (0.90, 0.98)	1.16 (1.13, 1.18)	1.11 (1.09, 1.13)
2004–2006	0.96 (0.93, 1.00)	1.12 (1.10, 1.14)	1.08 (1.07, 1.10)
2007–2009	Reference	Reference	Reference
2010–2013	1.02 (0.98, 1.06)	0.87 (0.85, 0.89)	0.90 (0.88, 0.92)
2013–2017	1.02 (0.97, 1.08)	0.78 (0.75, 0.80)	0.82 (0.80, 0.84)
Grouped frailty index:			
Fit	Reference	Reference	Reference
Mild frailty	1.23 (1.19, 1.27)	1.35 (1.33, 1.37)	1.33 (1.31, 1.35)
Moderate frailty	1.43 (1.36, 1.50)	1.68 (1.64, 1.73)	1.63 (1.59, 1.66)
Severe frailty	1.73 (1.62, 1.86)	2.15 (2.07, 2.22)	2.04 (1.98, 2.10)
Grouped consultation rate (contact/year):			
< 10	Reference	Reference	Reference
10‐ < 20	1.15 (1.11, 1.18)	1.17 (1.15, 1.19)	1.16 (1.15, 1.18)
20‐ < 30	1.30 (1.24, 1.37)	1.40 (1.36, 1.43)	1.38 (1.35, 1.41)
30+	1.34 (1.26, 1.43)	1.78 (1.73, 1.84)	1.69 (1.64, 1.74)
Harmful alcohol use	1.36 (1.25, 1.47)	1.69 (1.64, 1.75)	1.65 (1.60, 1.70)
BMI group:			
Underweight	1.31 (1.21, 1.40)	1.90 (1.83, 1.97)	1.77 (1.71, 1.83)
Normal	Reference	Reference	Reference
Overweight	0.84 (0.82, 0.86)	0.84 (0.83, 0.85)	0.84 (0.83, 0.85)
Obese	0.75 (0.72, 0.78)	0.95 (0.93, 0.97)	0.91 (0.90, 0.92)
Smoking status			
Never	Reference	Reference	Reference
Current	1.09 (1.05, 1.13)	1.76 (1.72, 1.79)	1.61 (1.58, 1.63)
Former	1.05 (1.02, 1.08)	1.15 (1.13, 1.17)	1.13 (1.11, 1.14)
History of:			
Chronic kidney disease	0.94 (0.91, 0.99)	1.12 (1.09, 1.14)	1.07 (1.05, 1.10)
Asthma	0.97 (0.94, 1.01)	0.94 (0.92, 0.96)	0.94 (0.93, 0.96)
Autoimmune disease	0.97 (0.93, 1.01)	1.01 (0.99, 1.03)	1.00 (0.98, 1.02)
COPD	0.94 (0.90, 0.98)	1.32 (1.29, 1.34)	1.24 (1.22, 1.27)
Depression (last 2 years)	1.56 (1.45, 1.68)	1.18 (1.14, 1.23)	1.25 (1.21, 1.30)
Hypertension diagnosis	0.89 (0.86, 0.91)	0.99 (0.98, 1.01)	0.97 (0.96, 0.98)
IHD diagnosis	0.98 (0.95, 1.01)	1.10 (1.08, 1.12)	1.08 (1.06, 1.09)
Severe immunosuppression	0.89 (0.84, 0.93)	1.63 (1.60, 1.67)	1.48 (1.45, 1.51)
Liver disease	0.93 (0.76, 1.14)	1.81 (1.69, 1.94)	1.68 (1.57, 1.80)
Stroke	1.30 (1.24, 1.36)	1.33 (1.29, 1.36)	1.32 (1.29, 1.35)
Traumatic brain injury	1.26 (1.10, 1.46)	0.92 (0.85, 1.00)	0.98 (0.92, 1.05)
HSV	1.00 (0.93, 1.07)	0.82 (0.79, 0.86)	0.86 (0.83, 0.89)
Uncontrolled diabetes (last 2 years)	1.15 (1.09, 1.21)	1.35 (1.31, 1.38)	1.31 (1.29, 1.34)

**Figure 4:**
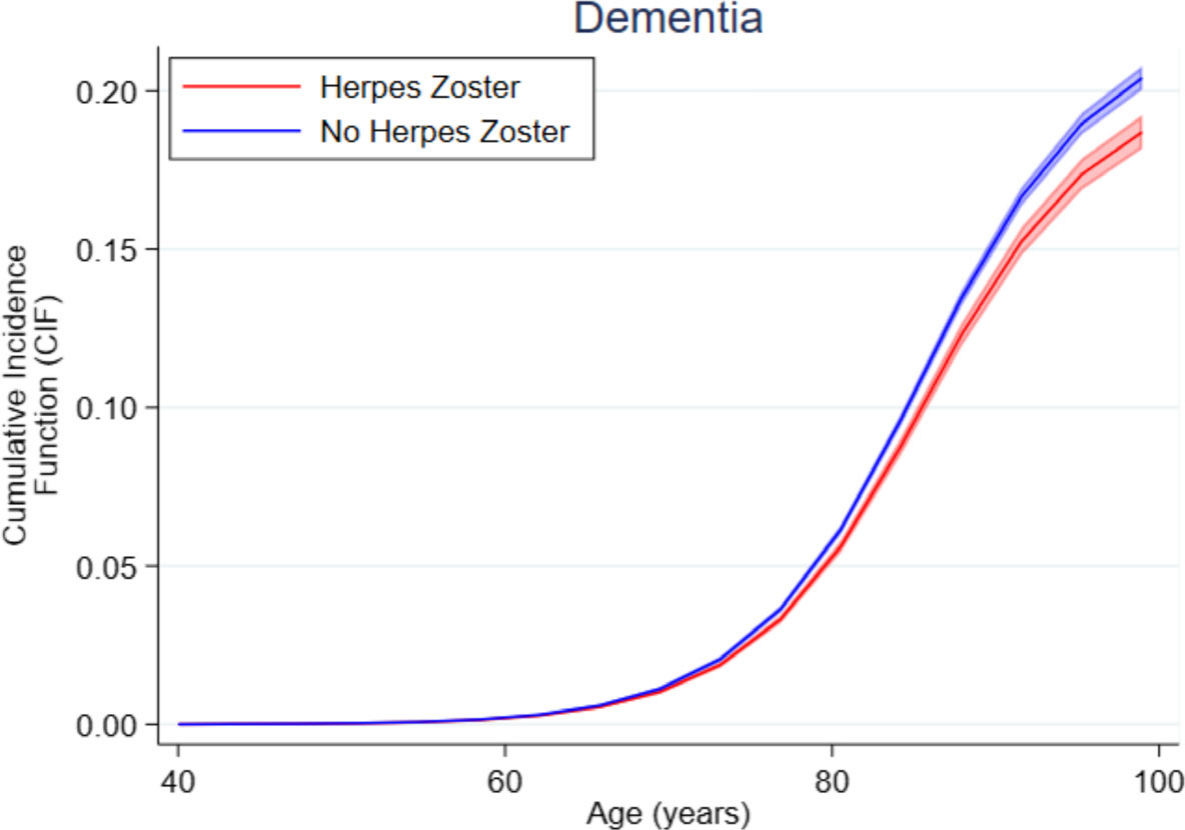
Cumulative cause‐specific incidence function for dementia, by herpes zoster status. [Colour figure can be viewed at wileyonlinelibrary.com]

Figure 4 shows estimated cumulative incidence function for dementia by HZ status; at 100 years, the estimated difference remains less than 2% points.

### Secondary analyses

i *Ophthalmic zoster*. Similar results were seen when restricting exposure to HZO only (adjusted HR 0.88, 95% CI 0.78–0.99) for dementia.
ii *Dementia subtypes*. There was no association between HZ and Alzheimer's disease (adjusted HZ 0.94, 95% CI 0.89–1.00), vascular dementia (adjusted HR 0.95, 95% C.I, 0.90–1.02) or other specified dementia subtypes (adjusted HR 0.91, 95% CI 0.77–1.07). A protective association was seen between HZ and dementia classified as mixed or unspecified (adjusted HZ 0.88, 95% CI 0.83–0.92).
iii *Interactions by sex, frailty and antiviral status*. Table 4 shows estimated HRs by sex, frailty and receipt of antiviral drugs. There was some evidence of an interaction of HZ with sex, with no association for males but a protective association for females. There was strong evidence of an interaction with frailty, with the protective association absent in the fittest patients at baseline and increasing with increasing frailty. There was no evidence of a difference in association according to antiviral treatment status.

**Table 4:** Estimated HRs for dementia, including interactions (individually) with sex, frailty and antivirals post‐exposure.

Variable	HR (95% CI)	Interaction p‐value
Sex		0.036
Female	0.89 (0.86, 0.93)	
Male	0.96 (0.91, 1.01)	
Grouped frailty index:		<0.001
Fit	0.97 (0.92, 1.02)	
Mild frailty	0.91 (0.86, 0.96)	
Moderate frailty	0.88 (0.82, 0.95)	
Severe frailty	0.82 (0.73, 0.92)	
Antivirals		0.774
No	0.91 (0.87, 0.96)	
Yes	0.92 (0.89, 0.95)	

### Model checking

There was no evidence against the proportional hazards assumption for the main exposure of interest, HZ, in any of these Cox models. However, the proportional hazards assumption was violated for several coefficients and stratifying the Cox model did not resolve all these problems. Stratifying by general practise, sex, calendar year, frailty, consultation rate, BMI group and history of stroke (the key variables leading to PH violations) made little difference to the hazard ratio for HZ (adjusted HR 0.94, 95% CI 0.90–0.98). When AICs for various model types were compared, the generalised gamma model, an accelerated failure time model which does not rely upon the proportional hazards assumption, fit best––see Table 5. This model estimates that the median time to dementia diagnosis is 1.009 times longer (i.e. increased by approximately 1%) in the HZ group compared to the unexposed group, which is consistent with findings from the proportional hazards models suggesting a small overall reduction in hazard of dementia diagnosis associated with HZ.

**Table 5:** AICs and estimated point estimates (Hazard ratio for PH models or time ratios for AFT models) for Herpes Zoster from various survival models.

Model	Model type	AIC	Point estimate (95% CI) exponentiated
Cox	PH	543,745.1	0.915 (0.887, 0.944)
Weibull	PH and AFT	43,607.56	0.908 (0.880, 0.936)
Log‐logistic	AFT	42,572.3	1.009 (1.006, 1.012)
Generalised gamma	AFT	42,327.88	1.009 (1.006, 1.012)

PH Proportional hazards; AFT accelerated failure time.

a Note: models all excluded general practise; this made little difference to the Cox model estimates.

## Discussion

### Summary of findings

We found no evidence that HZ was associated with an increased risk of subsequent dementia diagnosis in a large UK population‐based cohort. A small protective association between HZ and dementia was seen only for frail individuals and females, and for mixed/unspecified dementia. There was no difference by receipt of antiviral medications. HZ was not associated with a composite outcome of dementia or death. These findings were robust across various sensitivity analyses.

### Comparison with other studies

Our results were consistent with recent findings from other cohorts for example, from the SAIL Databank in Wales in which both diagnosed and treated individuals with HZ, as well as diagnosed and untreated individuals with HZ, had a 9%–10% lower risk of dementia diagnosis recording than individuals without HZ.^15^ In the same study, individuals with treated HZ from Denmark had a 10% lower dementia diagnosis risk, while there was no difference in dementia diagnoses in Germany among those diagnosed and treated for HZ.^15^ These findings contrast with studies using insurance claims data from Taiwan and Korea, three of which showed an increase in dementia risk after herpes zoster^11^, ^12^, ^13^ and two reporting a large reduction in dementia among those with HZ treated with antiviral medications.^12^, ^13^ Our sample size was at least twice as large as all previous studies, except for the Denmark cohort in which information on HZ diagnoses were not available and had to be inferred from antiviral dosage. Compared to the studies with contrasting findings, we adjusted for additional covariates; nevertheless, even without adjustment, we did not see differences in rates of dementia diagnoses between patients exposed and unexposed to HZ. We also used robust approaches to addressing missing covariate data which had little impact on the findings. Unique to our study was the use of different modelling approaches with varying underlying assumptions; consistency across these models adds confidence to our findings. We additionally identified that a small apparent protective association between HZ (with or without antiviral use) and dementia was confined to frail individuals and to females, and was seen only for mixed or unspecified dementia.

Differences between our study and findings from Taiwan and Korea may reflect methodological differences: previous studies either used future information to define covariates^12^ or the timing of covariate assessment was unclear;^11^, ^13^ in addition, these studies were unable to adjust for lifestyle factors and did not explore the role of frailty. The definitions of the dementia outcome in previous studies also differed for example, by requiring either several instances of a dementia code in outpatient data,^11^, ^12^ or 30 days of medication,^13^ dementia recording may have been more likely among those who sought care frequently, contributing to ascertainment bias. Finally, a contribution of genetic differences between populations cannot be excluded.

### Strengths and limitations

Strengths of our study include the large population size: we captured all patients with a record of HZ in a representative real‐world UK population over a 17‐year time period. We matched patients on age, sex, practise (which relates both to geographic region and socio‐economic status) and calendar time, and comprehensively controlled for confounders. We also carried out a range of sensitivity analyses to increase confidence in our estimates. Nevertheless, there are some limitations. HZ is potentially under‐recorded in EHRs: figures from the US suggest that around 90% of adults aged 50 years or over report seeking medical care for an episode of HZ.^20^ While younger patients who tend to have more mild disease are least likely to attend, these patients are also the least likely to have long‐term sequelae, including dementia, so the impact on our study is estimated to be small.

The positive predictive value (PPV) of a dementia diagnosis in routinely collected EHRs is relatively high^21^ and we focussed on all‐cause dementia as the primary outcome as the PPV of a dementia diagnosis varies by dementia subtype.^22^ However, in the UK only around two thirds of patients with dementia have a diagnosis recorded in primary care. We nevertheless saw similar results when expanding our outcome definition to include administrative as well as diagnostic and referral codes. These have been shown to improve detection of dementia in primary care records compared with diagnosis codes alone.^23^

Previous studies have identified characteristics associated with the likelihood of having a recorded dementia diagnosis in primary care among those with objective evidence of dementia.^24^ Diagnoses are more common among those with severe memory impairment and those living with a partner; conversely, people with extensive cardiovascular comorbidities, people aged 90 years or over and those living in residential care settings were less likely to be diagnosed. In our study, the apparent protective association between HZ and dementia was only apparent for frail individuals, who are likely to have many physical comorbidities. It is therefore possible that this group was disproportionately affected by under‐recording of dementia diagnoses, perhaps because of a focus on treating other physical conditions rather than prioritising memory clinic referrals. Finally, we controlled for health seeking behaviour based on mean GP consultations over the past 3 years, although if recording of dementia were differential by HZ status, this would typically tend to overestimate, rather than underestimate, associations between HZ and dementia.

Other limitations include the difficulty investigating risk factors for syndromes with long latency periods that potentially extend over decades: it is possible that, despite our longitudinal design, the follow‐up time was insufficient to capture any upstream effects of HZ that is, inducing early neuropathological changes that may eventually contribute towards a clinical dementia diagnosis. Nevertheless, the absence of evidence for any increase in dementia diagnosis with HZ, and apparent small protective association in certain population subgroups, suggests that HZ is unlikely to lead to a clinically significant increase in the risk of dementia diagnosis. Finally, as with any EHR‐based study there is potential for residual confounding by unmeasured factors such as those related to genetic risk. However, in the general population, genetic risk factors for HZ and dementia are likely to explain only a small proportion of the variation in risk.^25^, ^26^

### Implications

We found no evidence that HZ was associated with increased risk of dementia diagnosis in a large population‐based cohort from the UK. The apparent small protective association seen only in frail individuals may be partly explained by a competing risk of death in those individuals, in addition to preferential under‐recording of dementia among persons with multiple physical comorbidities. Despite epidemiological evidence and biological mechanisms linking HZ and acute cerebrovascular events, a lack of association between HZ and increased dementia risk may be unsurprising: HZ usually occurs as a single clinical episode provoking a transient immune response in immunocompetent individuals,^27^ which may not affect the long trajectory of dementia development.

Nevertheless, the relationship between persisting viruses and brain health over the life course remains underexplored. Future research should triangulate information across large, prospectively collected datasets from different populations to investigate associations further, taking careful steps to reduce biases for example, by including laboratory‐confirmed infection definitions, repeated regular assessments of outcome and comprehensive confounder control. Improved understanding of the complex interplay between infections, immune system modulation and chronic disease risk could help both to characterise susceptible individuals and identify routes for future intervention.

## Author Contributions

Conception and design of the study: CWG, EW, NP, JMB, SIS and LS. Acquisition and analysis of data: EW, SIS and JBH. Drafting manuscript and figures: CWG and EW. Reviewing manuscript for important intellectual content and approving the final version: CWG, EW, SIS, JBH, NP, JMB and LS.

## Conflict of Interest

SIS is now employed by the Clinical Practice Research Datalink (CPRD), a division of the UK Medicines and Healthcare products Regulatory Agency (MHRA), but the views expressed in this publication are his own and do not represent the official position of either the CPRD or the MHRA. All other authors report no conflict of interest.
